# Effect of combination of taurine and azelaic acid on antimelanogenesis in murine melanoma cells

**DOI:** 10.1186/1423-0127-17-S1-S45

**Published:** 2010-08-24

**Authors:** Ji Sun Yu, An Keun Kim

**Affiliations:** 1 Biochemistry Laboratory, College of Pharmacy, Sookmyung Women’s University, 52 Hyochangwon-gil, Yongsan-gu Seoul, 140-742, Korea

## Abstract

**Background:**

Pigmentation in human skin is an important defense mechanism against sunlight or oxidative stress. Despite the protective role of melanin, abnormal hyperpigmentation such as freckles and chloasma sometimes can be serious aesthetic problems. Because of these effects of hyperpigmentation, people have considered the effect of depigmentation. Azelaic acid (AZ) is a saturated dicarboxylic acid found naturally in wheat, rye, and barley. Previously, we showed that AZ inhibited melanogenesis. In this study, we investigated the antimelanogenic activity of combination of AZ and taurine (Tau) in B16F10 mouse melanoma cells.

**Methods:**

The mouse melanoma cell line B16F10 was used in the study. We measured melanin contents and tyrosinase activity. To gain the change of protein expression, we carried out western blotting.

**Results:**

We investigated that AZ combined with taurine (Tau) show more inhibitory effects in melanocytes than the treatment of AZ alone. AZ combined with Tau inhibited the melanin production and tyrosinase activity of B16F10 melanoma cells without significant cytotoxicity. Also inhibitory effects after treatment with these combined chemical are stronger than AZ alone on melanogenesis.

**Conclusions:**

These findings indicate that AZ with Tau might play an important role in the regulation of melanin formation and be useful as effective ingredients in antimelanogesis.

## Introduction

Melanin in human skin plays as a natural solar filter absorbing and reflecting most of the UV radiation passing through the layer. Increased production and accumulation of melanins describe a number of hyperpigmentary disorders such as melasma and postinflammatory hyperpigmentation (PIH) [1]. These hyperpigmentation can cause psychological and emotional concern. Recently, many efforts have been devoted to screening antimelanogenesis agents [2]. Antimelanogenesis can be achieved by controlling (i) the activity of tyrosinase, tyrosinase gene expression, tyrosinase related protein-1 (TRP-1) and tyrosinase related protein-2 (TRP-2); (ii) melanin and melanosome degradation and transfer to keratinocytes [3]. Recent studies suggested that another transcription factor, MITF (microphthalmia transcription factor) appear to play a regulatory role in early embryonic development of the pigment systems [4-6]. However, tyrosinase is the key role enzyme in melanin biosynthesis. Therefore, most of antimelanogenesis agents function specifically to reduce activity of this enzyme [7].

Azelaic acid (AZ) is a naturally occurring nonphenolic, saturated, nine-carbon dicarboxylic acid compound isolated from cultures *Pityrosporum ovale*. Initially, AZ was developed for treatment of topical acne. However, because of its inhibitory effect on tyrosinase, it has also been used to treat melasma and PIH [8]. In vitro studies show that AZ interferes with DNA synthesis and mitochondrial enzymes in abnormal melanocytes but does not affect normal melanocytes [2,8-11].

The aim of present study was to investigate the antimelanogenic activity of combination of AZ and antioxidant, taurine (Tau) in B16F10 mouse melanoma cells. Moreover, to get molecular insight into the inhibition of melanogenesis by combination of Tau and AZ, we investigated its effect on tyrosinase, TRP-1, TRP-2, MITF and phosphate ERK protein expression.

## Materials and methods

### Materials

AZ was purchased from Sigma-Aldrich (St. Louis, MO, USA). AZ was dissolved in dimethyl sulfoxide (DMSO) and the maximum concentration of DMSO was 0.1%. Dulbecco's Modified Eagle Medium (DMEM), dulbecco’s phosphate buffered saline (DPBS), fetal bovine serum (FBS), penicillin-streptomycin, and trypsin-EDTA were purchased from WelGENE (Daegu, South Korea).

### Cell lines and cell culture

The mouse melanoma cell line, B16F10, was obtained from Korean Cell Line Bank (KCLB, Seoul, South Korea). The cells were maintained in DMEM supplemented with 10% fetal bovine serum (FBS) and penicillin-streptomycin. Cultures were routinely maintained at 37°C in a humidified atmosphere of 5% CO_2._

### Determination of cytotoxicity

The effect of drugs on the proliferation rate/cytotoxicity of cancer cells was assessed by using a colorimetric MTT assay. Briefly, cells were grown in 96-well flat-bottomed plates in media with 10% FBS allowed attach overnight. Then media was removed and replaced with fresh media with various concentrations of drugs. At the end of the treatment, medium was replaced by MTT (2.5 mg/mL) solution and cells were incubated at 37°C. Following 4 hr of incubation, MTT solution was discarded formazan crystal was solubilized with DMSO. The optical densities were measured at 570 nm. Results were calculated as percentage of unexposed control.

### Measurement of melanin contents

B16F10 melanoma cells were seeded at a density of 2.5×10^5^ cells/60 mm culture dish. The cells were treated with AZ combined with Tau for 24 hr. The cells pellets were dissolved in 1 N NaOH at 60°C for 1 hr. The relative melanin content was determined by measuring the absorbance at 475 nm in ELISA reader.

### Tyrosinase activity assay

The tyrosinase activity was evaluated by measuring the rated of dopachrome formation of L-DOPA (L-3, 4-dihydroxyphenylalanine). After incubation of AZ with Tau for 24 hr, the cells were washed in ice-cold PBS twice and lysed in phosphate buffer (0.1 M, pH 6.8) containing 1% (w/v) Triton X-100. The cellular extract was clarified by centrifuged at 14000 rpm for 20 min.

### Western blot analysis

After treatment with drugs, cells were washed with phophate-buffered saline, harvested, and were lysed in RIPA buffer [50 mM Tris-HCl (pH 8.0) with 150 mM NaCl, 1.0% nonidet P-40, 0.5% sodium deoxycholate, and 0.1% sodium dodecyl sulfate] containing protease inhibitor and phosphatase inhibitor (Roche, Indianapolis, IN, USA). After centrifugation, the supernatant was separated and stored at -70 C until use. Protein concentration was quantified by using a protein assay kit (Bio-Rad, Hercules, CA). Equal amount of protein were subjected to sodium dodecyl sulfate-polyacrylamide gel electrophoresis and transferred to a polyvinylidene difluoride membrane. The membrane was blocked and incubated with primary antibody overnight in Tris-buffered saline with 0.2% Tween-20 and 2.5% nonfat dry milk (or 2.5% bovine serum albumin). The primary antibodies used in the study are as follows: ERK 1/2 and phospho-ERK 1/2 antibodies were purchased from Cell signaling (Danvers, MA, USA) and were used at a 1:1000 dilution. Phospho-ERK1/2 antibody detects endogenous levels of ERK 1/2 when phosphorylated Thr/Tyr of ERK1/2. TRP1, TRP2, tyrosinase and MITF antibodies were purchased from Santa Cruz Biotechnology (Santa Cruz, CA, USA) and used at a 1:500 dilution. Following three washes of 10 minutes with Tris-buffered saline with 0.2% Tween-20, blots were incubated with horseradish peroxidase-conjugated secondary antibody (Santa Cruz, CA, USA). The blots were washed again three times in Tris-buffered saline with 0.2% Tween-20 and visualized with an ECL advance detection system.

### Statistical analysis

All experiments were done at least 3 independent times and values were expressed as means ± S.D. Significant differences between groups were analyzed by using Student *t*-test. A P-value of < 0.05 was considered statistically significant.

## Results and discussion

### Effects of combination of Tau and AZ on viability

**Figure 1:**
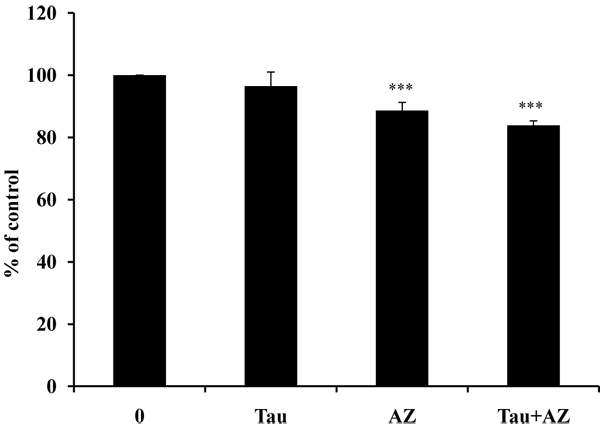
**Cell viability of Tau and AZ.** Cell viability of Tau and AZ was analyzed using the MTT assay. Cells were incubated with Tau (taurine 20 mM), AZ (azelaic acid 20 mM) and combination of Tau and AZ. Viability was calculated as the percent of control. The bars represent the mean values ± SD of triplicate. ^***^P < 0.001 versus control values.

Initially, the overall cytotoxic effect of combination of Tau and AZ in mouse melanoma cells was assessed by MTT assay. There were no growth inhibition effects of Tau 20 mM and showed slight growth inhibition effects at AZ 20 mM treatment (21.4%), and combination of Tau 20 mM and AZ 20 mM (27%) for 24 hr (Fig. 1). In further assessment, we used these concentrations.

### Inhibitory action of tyrosinase and melanin contents of B16F10 cells by combination of Tau and AZ

The antimelanogenesis can be helpful not only for cosmetic as whitening purposes but also for the treatment of abnormal pigmentation. Recently, tyrosinase inhibitor has been used as a antimelanogenesis agent because of its potential to inhibit dermal melanin formation [12].

**Figure 2:**
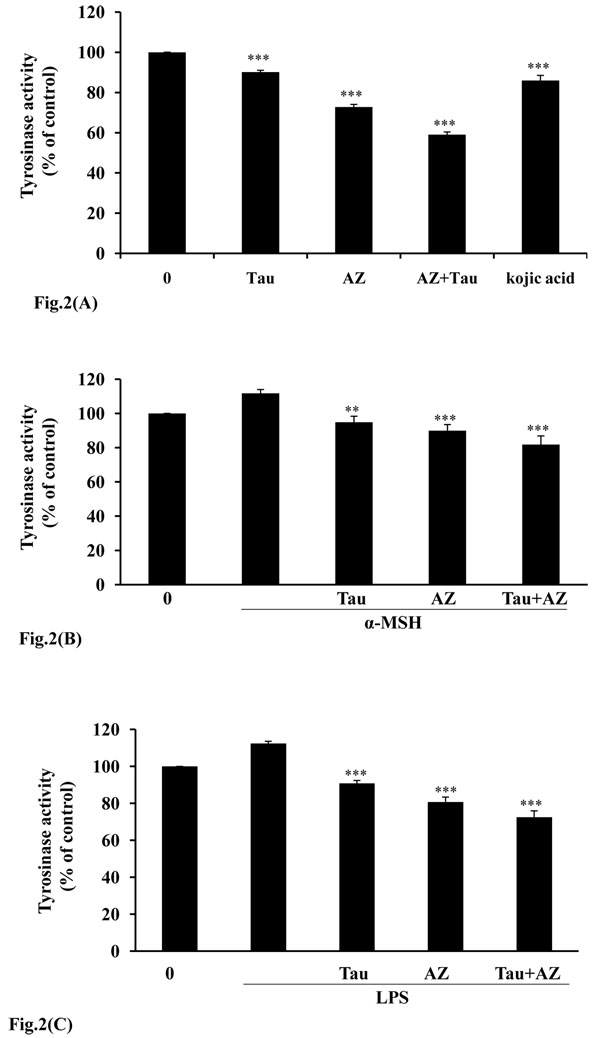
**Inhibitory effect of combination of Tau and AZ on tyrosinase enzyme activity.** (A) After incubation with Tau (20 mM), AZ (20 mM) or combination of Tau and AZ, tyrosinase activity was subsequently measured as described in the Material and Methods. B16F10 cells were incubated in the presence of (B) 100 nM of α-MSH or (C) 1 μg/ml of LPS and then treated for 24 hr with each drug. Tyrosinase activity was expressed as % of control. Results are the means of three independent experiments. The bars represent the mean values ± SD of triplicate. ^**^P< 0.01; ^***^P < 0.001 versus control values.

We investigated the inhibitory effect of combination of Tau and AZ on tyrosinase activity. As Fig. 2A indicates, AZ and Tau exhibit inhibition of tyrosinase activity. Especially, incubation with combination of Tau and AZ, tyrosinase activities were suppressed, showing 41% compare with control. To establish the relative efficacy of combination of Tau and AZ, we also compared with a well known antimelanogenic agents, kojic acid on tyrosinase activity. The inhibitory effect of combination of Tau and AZ was significantly less than to kojic acid. It has been known that melanin synthesis can be activated by α-melanocyte-stimulating hormone (α-MSH) and LPS [13,14]. In our study, we used α-MSH and LPS as melanogenic inducers. B16F10 cells were incubated in the presence of 100 nM of α-MSH or 1 μg/ml of LPS and then treated for 24 hr with each drug. As shown in Fig 2B and 2C, Tau, AZ and combination of Tau and AZ effectively inhibited tyrosinase activities as compared to the α-MSH or LPS treated groups.

**Figure 3:**
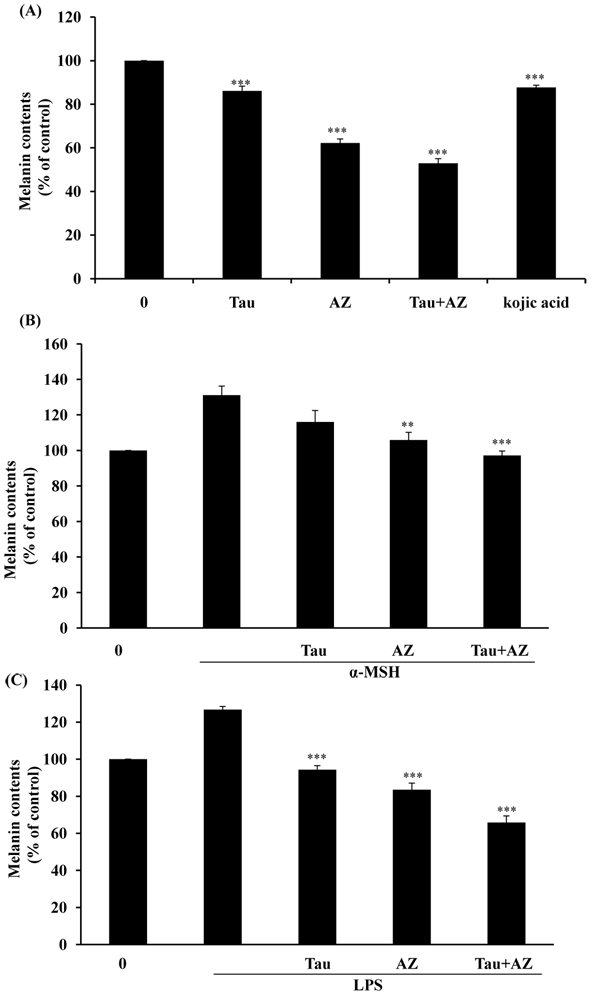
**Melanin contents in B16F10 cells.** (A) After incubation with Tau (20 mM), AZ (20 mM) or combination of Tau and AZ, melanin content was subsequently measured as described in the Material and Methods. B16F10 cells were incubated in the presence of (B) 100 nM of α-MSH or (C) 1 μg/ml of LPS and then treated for 24 hr with each drug. Total melanin content was expressed as % of control. Results are the means of three independent experiments. The bars represent the mean values ± SD of triplicate. ^**^P< 0.01; ^***^P < 0.001 versus control values.

To get evidence on the involvement of combination of Tau and AZ in melanogenesis, we analyzed the inhibition of melanin production in B16F10 cells. As exhibited in Fig. 3A, treatments with Tau, AZ and combination Tau and AZ, the melanin contents were decreased: 24 % of Tau, 37% of AZ, 49% of combination of Tau and AZ compared to control. We also examined inhibitory effects of melanin contents in α-MSH or LPS -induced B16F10 melanoma cells. Results showed that melanin synthesis was effectively inhibited in α-MSH or LPS-induced B16F10 melanoma cells (Fig. 3B and 3C). These results indicated that combination of Tau and AZ exhibited more powerful inhibitory effects on tyrosinase activity and melanin production compared to single treatment of Tau or AZ.

### Effects of combination of Tau and AZ on the expression of tyrosinase, TRP -1 and TRP-2

**Figure 4:**
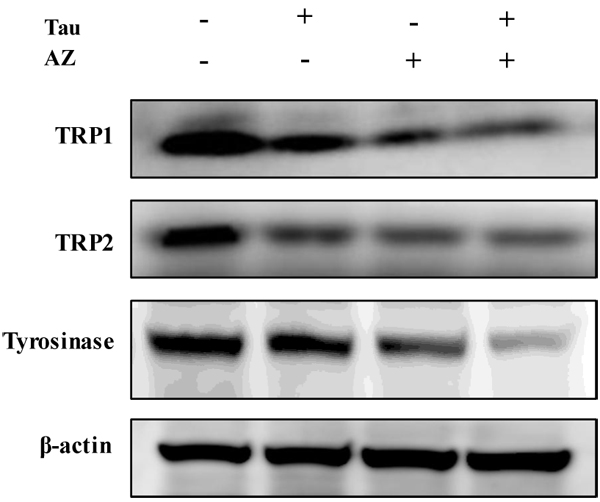
**Inhibitory effect of combination of Tau and AZ on tyrosinase, TRP1 and TRP2.** Cells were treated with each drug. Cell lystates were examined by western blotting analysis using TRP1, TRP2 and tyrosinase antibodies

The tyrosinase gene family including tyrosinase, TRP-1 and TRP-2 has been determined to play an important role in the regulation of melanogenesis [15]. Next, to know the effects of combination of Tau and AZ on tyrosinase or other melanogenic enzyme like a TRP-1 and TRP-2 expression, western blot analysis was performed on to these proteins. There was reduction of tyrosinase, TRP-1, TRP-2 expression with single treatment of Tau or AZ and combination of Tau and AZ compared with control (Fig. 4). From the western blot analysis, reduce of melanin contents and tyrosianse activity were associated with attenuated amount of tyrosinase, TRP1 and TRP2 protein levels.

### Effects of combination of Tau and AZ on MITF expression

To gain further molecular insight into the inhibition of melanogenesis, we investigate the effects on ERK 1/2 and melanocyte-specific transcription factor (MITF) molecules by combination of Tau and AZ.

**Figure 5:**
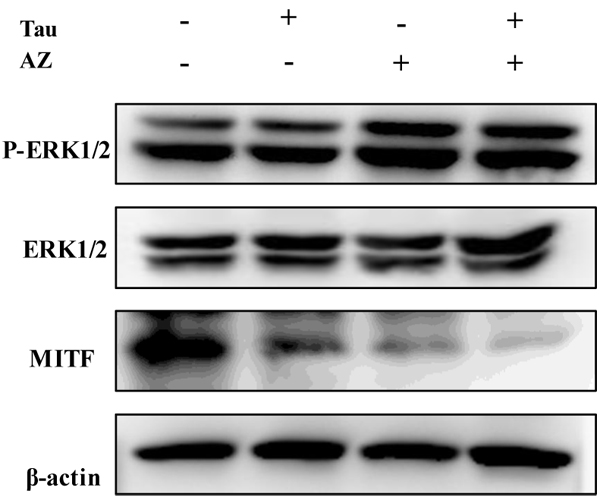
**Effect of combination of Tau and AZ on activation of ERK and MITF protein.** Cells were treated with each drug. Cell lystates were examined by western blotting analysis using total and phospho ERK and MITF antibodies.

MITF is one of the pivotal transcriptional regulators and is associated with the pigmentation, proliferation and survival of melanocytes [16]. MITF effectively transactivates the expression of tyrosinase and its related genes by binding to their common promoters [17]. We examined the MITF expression after single treatment of Tau or AZ and combination of Tau and AZ by western blot. As shown in Fig. 5, there was reduction of MITF level by single treatment of Tau or AZ, and reduction of treatment of combination of Tau and AZ was especially dramatic.

Several studies have reported that ERK is an important regulator of melanogenesis because ERK activation phosphorylates MITF and its subsequent degradation by ubiquitination and degradation [18,19]. To investigate the effects of combination of Tau and AZ on ERK activation, western blot analysis was carried out. Combination of Tau and AZ stimulated the phosphorylation of ERK (Fig. 5). These results indicated that antimelanogenic activity of combination of Tau and AZ was associated with suppression of MITF and activation of ERK phosphorylation.

## Conclusions

In summary, we have demonstrated that combination of Tau and AZ inhibited melanin production and tyrosinase activity. Western blotting data indicated that the antimelanogenic activity of treatment with combination of Tau and AZ is probably due to suppression of tyrosinase, TRP1, TRP2 and MITF and increase of ERK activation in B16F10 mouse melanoma cells. Thus, our data suggested that combination of Tau and AZ may potentially be used in development of depigmenting agents.

## Competing interests

No competing financial interests exist.

## Authors' contributions

JS Yu carried out the functional study and drafted the manuscript. AK Kim helped to draft the manuscript and participated in the design of the study. All authors read and approved the final manuscript
